# N6-Methyladenosine Methyltransferase METTL14-Mediated Autophagy in Malignant Development of Oral Squamous Cell Carcinoma

**DOI:** 10.3389/fonc.2021.738406

**Published:** 2021-11-24

**Authors:** Fang Wang, Yue Zhu, Hongshi Cai, Jianfeng Liang, Wenjin Wang, Yan Liao, Yadong Zhang, Cheng Wang, Jinsong Hou

**Affiliations:** ^1^ Department of Oral and Maxillofacial Surgery, Guanghua School of Stomatology, Hospital of Stomatology, Sun Yat-Sen University, Guangzhou, China; ^2^ Guangdong Provincial Key Laboratory of Stomatology, Sun Yat-Sen University, Guangzhou, China; ^3^ Department of Oral Surgery, Ninth People’s Hospital, Shanghai Jiao Tong University School of Medicine, Shanghai, China

**Keywords:** oral squamous cell carcinoma, autophagy, RNA methylation, tumorigenesis, methyltransferase-like 14

## Abstract

N6-methyladenosine (m^6^A) is the most abundant internal mRNA modification in eukaryotes and is related to stability, localization, or translation efficiency in tumorigenesis. Autophagy plays an important role in the occurrence and development of tumours. However, the relationship between m^6^A and autophagy remains unclear. In this study, we used a rapamycin-induced autophagy model of oral squamous cell carcinoma (OSCC) cells, and observed increased m^6^A RNA methylation. When autophagy was activated, the methyltransferase-like 14 (METTL14) expression was upregulated and influenced the proliferation, migration, and invasiveness of OSCC cells. Through meRIP-seq and RNA-seq analysis, we found that METTL14 directly combined with eukaryotic translation initiation factor gamma 1 (eIF4G1) mRNA and decreased its RNA stability. According to the dual-luciferase reporter and mutagenesis assay, the mutated site 1 of exon 11 of eIF4G1 is the key target of METTL14. Knockdown of the main m^6^A binding protein YTHDF2 may rescue the shortened half-life of *eIF4G1* mRNA induced by METTL14 overexpression. Furthermore, an *in vivo* tumour xenograft model confirmed that a high METTL14 expression can effectively reduce OSCC growth. Additionally, using clinical samples, we found that patients with advanced or moderately/poorly differentiated tumours exhibited lower METTL14 levels. Taken together, our results revealed that METTL14 mediated eIF4G1 expression *via* m^6^A modification and regulated autophagy levels and biological functions in OSCC. Our findings not only expand our understanding of the correlation between autophagy and RNA methylation in tumorigenesis but also present an opportunity to develop new therapeutic options.

## Introduction

Oral cancer is one of the most common malignant tumours in the head and neck region. Oral squamous cell carcinoma (OSCC) is the most common subtype of oral cancer, accounting for approximately 90% of cases (1, 2). This type of carcinoma is highly invasive, often infiltrating and developing rapidly. Due to the frequent mobility of the tongue, facial muscles, and temporomandibular joint, and the abundant blood circulation and lymphatic drainage system in oral and maxillofacial regions, OSCC is prone to early cervical lymph node metastasis and distant metastasis (3, 4). Despite the rapid development and progress in imaging, surgery, radiotherapy, and multidisciplinary treatments, the five-year overall survival rate of patients with OSCC is approximately 50%, which is significantly lower than that of patients with other tumours (5, 6). The main reason for the poor prognosis of patients with OSCC is that OSCC cells have high invasion and metastatic abilities at an early stage (7). Therefore, it is of great clinical significance to study the invasion and metastasis molecular mechanisms of OSCC, which could aid in the development of new methods for early diagnosis and treatment.

Autophagy, an important process through which cells degrade endogenous substrates using lysosomes, maintains homeostasis by degrading intracellular macromolecules and removing damaged organelles (8–11). Autophagy is an important regulatory mechanism of the growth, differentiation, and death of tumour cells, and is closely related to the occurrence and development of tumours, but autophagy levels and functions differ between tumours (12, 13). Our previous studies have shown that the autophagy level of OSCC is significantly lower than that of adjacent non-tumour tissues, and is closely related to the clinical stage and prognosis of OSCC. Rapamycin can induce autophagy and inhibit cell migration, invasion, and proliferation in OSCC (14, 15). These results suggest that the malignant progression of OSCC is closely related to changes in autophagic activity, but the core mechanism regulating autophagy activity remains unclear.

We found that the methylation level of N6 methyladenine (m^6^A) increased in a rapamycin-induced autophagy cell model of OSCC, indicating that m^6^A RNA methylation may be involved in the activation of autophagy in OSCC cells. m^6^A RNA is a methylated nucleoside occurring at the sixth nitrogen atom of adenine and is the most abundant form of eukaryotic mRNA expression (16). Recent studies have shown that m^6^A affects malignant tumour progression by regulating the proliferation, differentiation, and apoptosis of tumour cells (17–21), but the relationship between m^6^A and autophagy is not entirely clear. In our previous studies, we have discovered the role of m^6^A demethylation by FTO in regulating autophagy (22). In this study, we further explored the specific molecular mechanism of m^6^A RNA methyltransferase METTL14 for regulating autophagy in OSCC.

## Materials and Methods

### OSCC Patient Samples

Eighty-two OSCC tissues and nineteen adjacent non-cancerous tissues (ANCT) were collected from the Hospital of Stomatology, Sun Yat-Sen University. The pathological diagnosis and staging of the patients were determined according to the 8^th^ American Joint Committee on Cancer Staging System. Fresh samples were fixed in paraformaldehyde for immunohistochemical analysis after surgical resection. This study was approved by the hospital’s ethics committee (KQEC-2020-15) and conforms to the Declaration of Helsinki.

### Cell Lines, Culture, and Drug Treatment

Human OSCC cell lines (HSC3, HN6, CAL33) were obtained from the American Type Culture Collection. HSC3 and CAL33 cells were cultured in Dulbecco’s modified Eagle’s medium (DMEM; Gibco, Waltham, MA, USA) containing 10% foetal bovine serum (FBS; Gibco). The HN6 cells were maintained in DMEM/Ham’s F12 medium (Gibco) supplemented with 10% FBS. For starvation treatment, the cells were incubated in glutamine-free DMEM without FBS (Gibco, USA). All cells were incubated at 37°C under a humidified atmosphere with 5% CO_2_. Rapamycin (100 nM) was added to the culture medium to induce autophagy, and 10 mM 3MA was used to inhibit autophagy.

### Total RNA m^6^A Quantification

The total mRNA m^6^A level was measured using an m^6^A RNA methylation quantification kit (Abcam, Cambridge, MA, USA). Each assay well contained 200 ng sample RNA and the corresponding dilution concentration gradient of the detection antibody. The m^6^A level was colorimetrically quantified by measuring the absorbance at 450 nm.

### RNA Extraction, qRT-PCR, and RNA-Seq

Total RNA was isolated using RNAzol^®^ RT (RN190; Molecular Research Center, Cincinnati, OH, USA). Total RNA (1 μg) was reverse-transcribed to complementary DNA using the PrimeScript RT reagent kit (RR036A; Takara Bio, Kusatsu, Shiga, Japan). The qPCR reactions were performed using SYBR Green Master Mix (11201ES08; Yeasen, Shanghai, China) and amplified using the LightCycler 96 System (Roche, Basil, Switzerland) according to the manufacturer’s instructions. The relative RNA expression of each gene was calculated using the 2^−△△Ct^ method normalized to *GAPDH*. The primer sequences used are as follows: eIF4A1- forward 5′-GAAGCTCCCCACATCATC-3′; eIF4A1- reverse 5′-ACGGCTTAACATTTCGTCA-3′; eIF4G1- forward 5′-CCCGAAAAGAACCACGCAAG-3′; eIF4G1- reverse 5′-TTCCCCTCGATCCTTATCAGC-3′.

### Western Blot Analysis

Cells were lysed using RIPA buffer (CW2333S; CWbio, Beijing, China) supplemented with a protease inhibitor cocktail set I (539131; Millipore, Burlington, MA, USA). Western blot was performed according to a standard protocol as previously described (15). Primary antibodies included anti-mTOR (#2983), anti-METTL3 (#86132), anti-WTAP (#41934), anti-FTO (#31687), anti-ALKBH5 (#80283), anti-LC3B (#4599), anti-eIF4G1 (#2858) (1:1 000, Cell Signaling Technology, Danvers, MA, USA), anti-METTL14 (#ab220030, 1:1 000, Abcam, Cambridge, UK), and anti-GAPDH (#G5262, 1:1 000, Sigma-Aldrich, St. Louis, MO, USA). Western blot band intensity was quantified using ImageJ 1.8.0 (https://imagej.nih.gov/ij/download.html).

### Lentiviral Transfection and Transient Transfection

Lentiviral oeRNAs of METTL14 and an empty vector as control (OECtrl) were cloned into a pLVoeRNA-EGFP (2A) lentiviral vector (GeneChem, Shanghai, China). The lentiviral packaging system with pseudoviral particles was delivered into HSC3 and CAL33 cells according to the manufacturer’s instructions. Small interfering RNAs (siRNAs), such as siMETTL14 (#1 5′-GCTGGACTTGGGATGATATTA-3′; #2 5′-GAACCTGAAATTGGCAATATA-3′), siYTHDF2 (#1 5′-GCCCAAUAAUGCAUAUACUTT-3′; #2 5′-GCUCUGGAUAUAGUAGCAATT-3′), and siYTHDC2 (#1 5′-GCGACUCAACAAUGGCAUATT-3′; #2 5′-GGAUUUGAUCAUGCAUCUUTT-3′), were used for METTL14, YTHDF2, and YTHDC2 knockdown, respectively. Lipofectamine 3000 (L3000-015; Invitrogen, Waltham, MA, USA) was used for lentiviral transfection, while Lipofectamine RNAiMax (13778150; Invitrogen) was used for transient transfection.

### Transmission Electron Microscopy (TEM)

Trypsin-digested cells were washed twice with phosphate buffer, fixed in 2.5% glutaraldehyde for 2 h and 1% osmium tetroxide for another 2 h, dehydrated in ethanol and acetone, and embedded in albite. Ultrathin sections (60 nm) were stained with 1% uranyl acetate, and images were observed using a Philips CM10 transmission electron microscope (Philips, Amsterdam, the Netherlands).

### MeRIP-Seq and MeRIP-qPCR

Total RNA was extracted from HSC3 and CAL33 cells treated with or without 100 nM rapamycin for three days, and the Magna Me-RIP m^6^A Kit (Merck Millipore, Burlington, MA, USA) with an anti-m^6^A antibody was used to perform immunoprecipitation. After immunoprecipitation, part of the target RNA was analysed using high-throughput sequencing (Epibiotek, Guangzhou, China), while the rest was quantified using RT-qPCR.

### RNA Stability Assay

HSC3 and CAL33 cells transfected with OEMETTL14, OEMETTL14+siYTHDF2, OEMETTL14+siYTHDC2, and OECtrl were treated with 5 μg/mL actinomycin D (a9415; Sigma). At 0, 6, 12, and 18 h, RNA was extracted using RNAzol^®^ RT for qRT-PCR. The half-life (t_1/2_) of the *eIF4G1* mRNA was calculated and normalized against *GAPDH.*

### Luciferase Reporter Assay

Cells were transfected with psiCHECK2, psiCHECK2-WT, psiCHECK2-Mut1, psiCHECK2-Mut2, or psiCHECK2-Mut3 in a 6-well plate. Six hours after transfection, each cell line was seeded into a 96-well plate. The dual-luciferase reporter assay system (Promega, Madison, WI, USA) was used after 24 h of incubation according to the manufacturer’s instructions. *Renilla* luciferase activity was normalized against firefly luciferase activity to evaluate reporter translation efficiency.

### Cell Proliferation Assay

HSC3 and CAL33 cells transfected with siMETTL14 or siCtrl with or without rapamycin induction were seeded in triplicate into 96-well plates at a density of 2 × 10^3^ cells per well. Cell proliferative ability was assessed at 0, 24, 48, 72, and 96 h using the Cell Counting Kit-8 (CCK-8) (40203ES80; Yeasen). In each well, 100 μL of 10% CCK8 reagent with cell suspension were added and incubated for 1 h at 37°C in a 5% CO_2_ atmosphere. The absorbance was measured at 450 nm using a microplate reader (Bio-Rad, Hercules, CA, USA), and the growth curve was delineated.

### Cell Migration and Invasion Assay

HSC3 (5 × 10^4^) or CAL33 (8 × 10^4^) cells were seeded in the upper chambers with 200 μL of FBS-free media and incubated with 10% FBS culture medium in the lower chambers (Corning, Beijing, China). *In vitro* cell migration and invasion were measured using the BD BioCoat™ system (BD Biosciences, San Jose, CA, USA). For the invasion assay, Transwell inserts coated with 0.33 mg/mL Matrigel (354234; Corning) were used. After 24 h of incubation, cells that migrated to or invaded the opposite side of the inserts were fixed, stained with haematoxylin, and counted using a microscope (100×, five random fields per chamber).

### Tumour Xenograft

Specific pathogen-free (SPF) female NOD/SCID mice (5–6 weeks old) were randomly distributed into two groups: the OECtrl group and the OEMETTL14 groups. Phosphate buffer (200 μL) containing approximately 5 × 10^7^ HSC3 or CAL33 cells was subcutaneously injected into the inner thigh of each mouse. Tumour volume (mm^3^) was measured every two days using a calliper and calculated using the following formula: V = (length × width^2^/2). The mice were euthanized two weeks after injection, and the tumour xenografts were harvested, photographed, weighed, and fixed. All animal studies were conducted with the approval of the Sun Yat-sen University Institutional Animal Care and Use Committee.

### Immunohistochemistry (IHC) Assay

Clinical samples from OSCC patients were fixed with paraformaldehyde and embedded in paraffin to prepare 4-μm sections. Immunohistochemistry staining was performed according to our previous study. Tissue sections were incubated with primary antibodies against METTL14 (1:1000, CL4252; Abcam) overnight at 4°C. The IHC score was evaluated according to the staining intensity of METTL14 and graded into high or low expression groups using the cut-off point of 150.

### Statistical Analysis

Statistical analyses were performed as described in the figure legends using GraphPad Prism 7.0 (GraphPad Software, LLC, San Diego, CA, USA). All descriptive values are presented as mean ± SD or SE. Statistical differences between the two groups were analysed using a two-tailed unpaired or paired Student’s t-test; for multiple groups, ANOVA with bonferroni’s multiple comparison tests were performed. The association between METTL14 expression and clinicopathological features in OSCC patients was determined using Fisher’s exact test. A *p*-value < 0.05 was considered statistically significant.

## Results

### Autophagy Activation Promoted the Upregulation of METTL14 Expression in OSCC Cells

**Figure 1:**
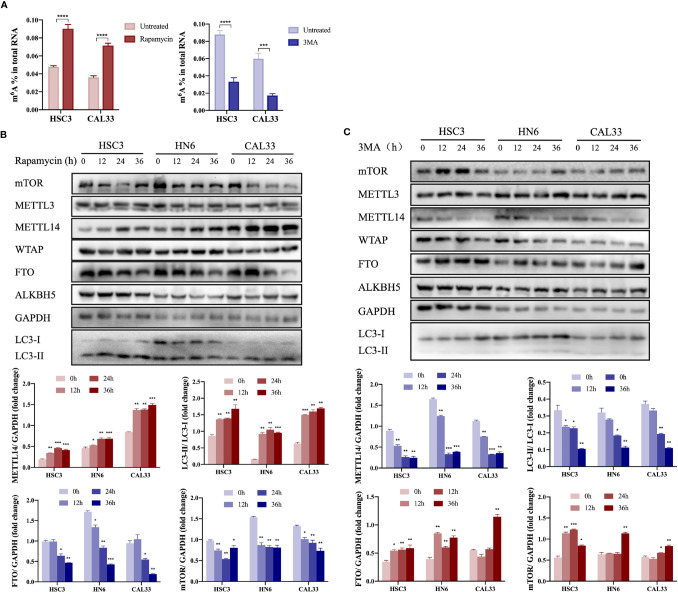
Autophagy activation promoted upregulation of METTL14 expression in OSCC cells. **(A)** In the autophagy cell model induced by rapamycin (100 nM rapamycin for three days) or inhibited by 3MA (10 mM 3MA for 24 h), the m^6^A level in OSCC cells was determined using an m^6^A RNA methylation quantification kit (Student’s *t*-test). **(B)** HSC3, HN6, and CAL33 cells were exposed to 100 nM rapamycin for 0, 12, 24, and 36 h to induce autophagy. Thereafter, mTOR, METTL3, METTL14, WTAP, FTO, ALKBH5, LC3-I, and LC3-II expression levels were determined using western blot (Student’s *t*-test). **(C)** HSC3, HN6, and CAL33 cells were exposed to 10 mM 3MA for 0, 12, 24, and 36 h to inhibit autophagy. Thereafter, mTOR, METTL3, METTL14, WTAP, FTO, ALKBH5, LC3-I, and LC3-II expression levels were determined using western blot (Student’s *t*-test). (*p* or adjusted-*p* *< 0.05, **< 0.01, ***< 0.001, ****< 0.0001).

When autophagy was induced by rapamycin or inhibited by 3MA *in vitro*, we found that m^6^A modification was upregulated or downregulated, respectively, in OSCC cells (**Figure 1A**), suggesting that m^6^A methylation may be involved in autophagy activation in OSCC cells. In autophagy-activated cells (treatment with rapamycin or under starvation conditions), m^6^A methyltransferase METTL14 expression was upregulated (**Figure 1B** and **Supplementary Figure S1**), while demethyltransferase FTO expression was downregulated (**Figure 1B**). However, we obtained the opposite result in autophagy-inhibited cells (treatment with 3MA) (**Figure 1C**), suggesting that METTL14 and FTO may act as key enzymes affecting m^6^A expression during autophagy activation. In a previous study, we demonstrated the inhibitory effect of FTO on autophagy (22). Therefore, the present study focused on the effect of METTL14 on autophagy.

### METTL14 Promoted Autophagy in OSCC Cells

**Figure 2:**
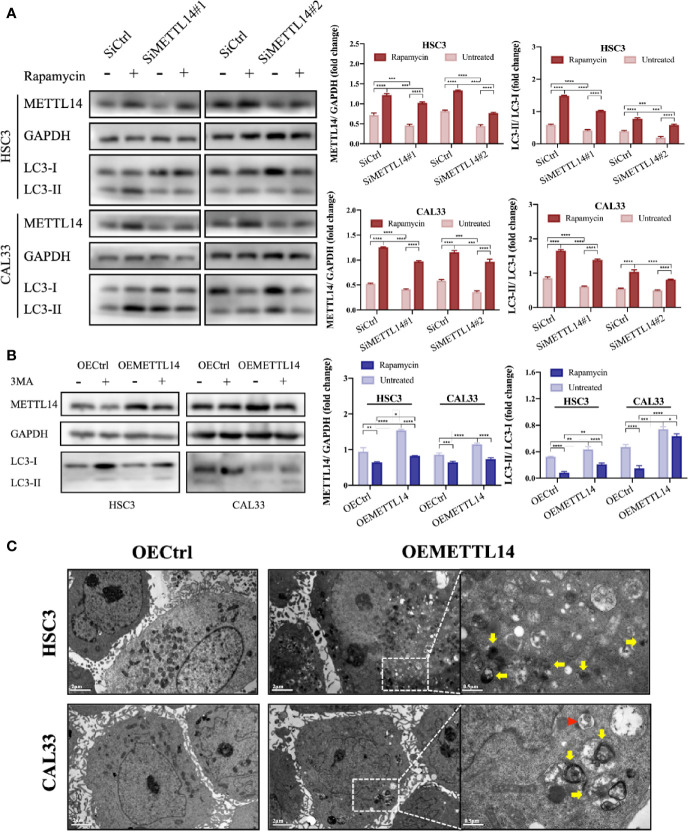
METTL14 promoted autophagy of OSCC cells. **(A)** METTL14 siRNAs were transiently transfected into HSC3 and CAL33 cells, with or without rapamycin. Western blot was used to detect the expression of METTL14, LC3-I, and LC3-II. **(B)** Plasmids containing lentiviral oeRNAs of METTL14 were transfected into HSC3 and CAL33 cells, with or without 3MA. Western blot was used to detect the expression of METTL14, LC3-I, and LC3-II. **(C)** Transmission electron microscopy showed the formation of autophagosomes (red arrows) and autophagolysosomes (yellow arrows) in OSCC cells overexpressing METTL14. (*p* or adjusted-*p* *< 0.05, **< 0.01, ***< 0.001, ****< 0.0001).

In order to elucidate the effect of METTL14 on autophagy in OSCC cells, we silenced or overexpressed METTL14 and found that the autophagy level (LC3B-II/LC3B-I) decreased after silencing METTL14. At the same time, we used rapamycin to induce autophagy for a rescue experiment and found that the autophagy level increased (**Figure 2A**). The results were contrary to the above situation when METTL14 was overexpressed and 3MA was used to inhibit autophagy (**Figure 2B**). In addition, TEM showed that METTL14 overexpression promoted autolysosome formation (**Figure 2C**). These results suggest that METTL14 promotes autophagy.

### METTL14 Regulated the Stability of *eIF4G1* mRNA Through Methylation

**Figure 3:**
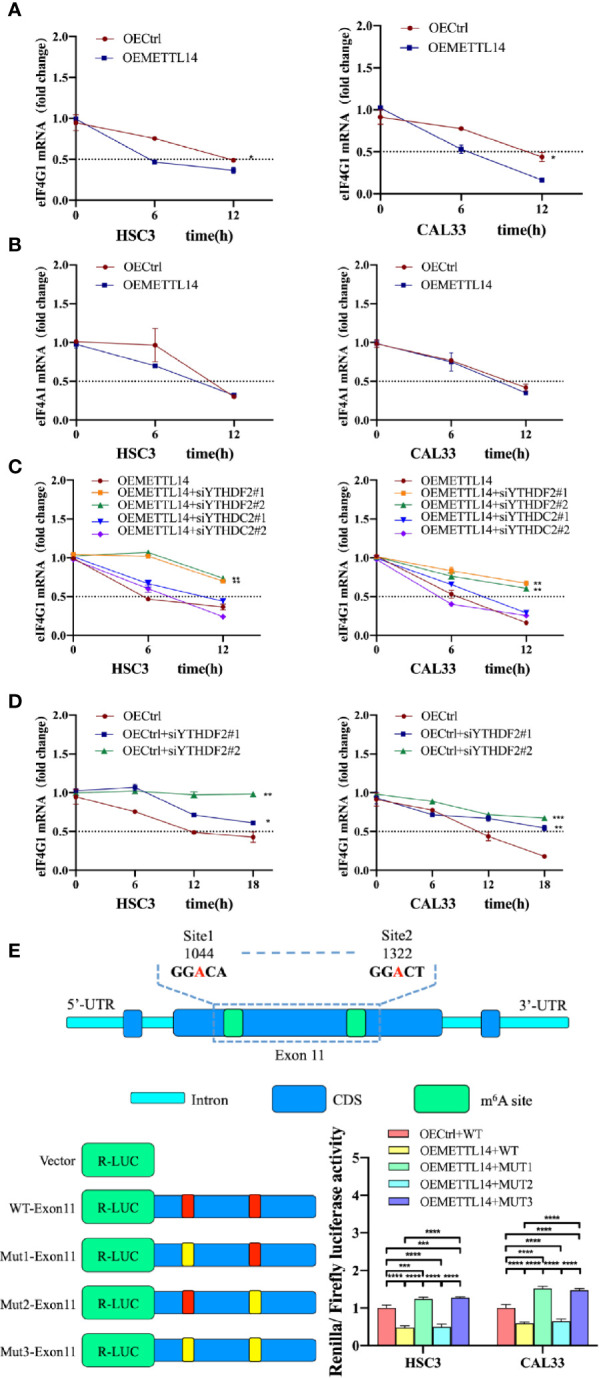
METTL14 regulated the stability of eIF4G1 mRNA *via* methylation. RNA stability assays showed *eIF4G1* **(A)** and *eIF4A1* **(B)** mRNA half-lives (t_1/2_) in OEMETTL14 cells compared with OECtrl cells. YTHDF2 or YTHDC2 were silenced based on OEMETTL14 **(C)** or OECtrl **(D)**, followed by the detection of *eIF4G1* mRNA t_1/2_. **(E)** Dual-luciferase reporter and mutagenesis assays were used to determine key targets on eIF4G1 for METTL14 to regulate degradation. (*p* or adjusted-*p* *< 0.05, **< 0.01, ***< 0.001, ****< 0.0001).

Eukaryotic translation initiation factor 4 gamma 1 (eIF4G1) is an important translation factor in eukaryotic organisms, promoting mitochondrial activity and cell proliferation while inhibiting autophagy (23). Through meRIP-seq and RNA-seq analysis (22), we found that *eIF4G1* mRNA was downregulated and m^6^A level was upregulated in the rapamycin-induced autophagy cell model. We speculated that m^6^A triggers the degradation of *eIF4G1* mRNA. To verify this, we detected the stability of *eIF4G1* mRNA in OEMETTL14 stable cells and control cells. The results showed that the half-life of *eIF4G1* mRNA was shorter in OEMETTL14 cells (**Figure 3A**), indicating that METTL14 overexpression reduced *eIF4G1* mRNA stability. eIF4A1 is another important component of eukaryotic translation initiation factor complexes (23). In order to further test the specific effect of METTL14 on *eIF4G1* mRNA, we tested the stability of *eIF4A1* as a positive control. Results showed that there was no significant difference in the stability of *eIF4A1* mRNA (**Figure 3B**), suggesting that *eIF4G1* mRNA is a METTL14 target but not *eIF4A1* mRNA.

A previous study described YTHDF2 as the primary m^6^A binding protein that promotes the degradation of mRNA (24). At the same time, another study found that YTHDC2 also promotes the degradation of mRNA (25). Therefore, we knocked down YTHDF2 or YTHDC2, based on METTL14 overexpression, and detected the stability of *eIF4G1* mRNA. The results showed that in OEMETTL14 cells, the degradation rate of *eIF4G1* slowed down when YTHDF2 was knocked down, but there was no significant change when YTHDC2 was knocked down (**Figure 3C**). Likewise, in the negative control experiment using OECtrl cells, YTHDF2 knockdown could also slow down the degradation rate of *eIF4G1* mRNA (**Figure 3D**). These results indicate that METTL14 overexpression promotes *eIF4G1* mRNA degradation, which depends on the expression of YTHDF2 rather than YTHDC2.

According to meRIP-seq data, there are multiple m^6^A peaks in the CDS region of eIF4G1 DNA. The m^6^A peak in exon 11 (CHR3: 184 314 495 to 184 335 358) was upregulated in both groups (**Supplementary Figure S2**). We identified two “GGAC” motifs in this region. We performed a dual luciferase reporter gene experiment to further clarify whether METTL14 regulates eIF4G1 through the above base sites. The constructed plasmids included WT-exon11 (wild type), mut1-exon11 (mutated site 1, GGAC mutated to GGTC), mut2-exon11 (mutated site 2), and mut3-exon11 (mutated site 2). The results showed that, compared with the control cells transfected with wt-exon11, the luciferase activity of METTL14 overexpressed stable cells transfected with WT-exon11 and mut2-exon11 decreased, while the luciferase activity of METTL14 overexpressed stable cells transfected with mut1-exon11 and mut3-exon11 increased (**Figure 3E**). The results showed that eIF4G1 degradation was inhibited by a mutation at site 1 (1044) of exon 11 or at any two sites at the same time, thus enhancing luciferase activity; however, there was no significant difference between mutant site 2 (1322) and the wild type without mutation, indicating that site 2 was not the catalytic site of METTL14 for eIF4G1 methylation. Therefore, site 1 of eIF4G1 exon 11 is the key target for the modification enzyme to regulate degradation.

### METTL14 Regulates OSCC Autophagy and Biological Functions by Mediating eIF4G1 Expression

**Figure 4:**
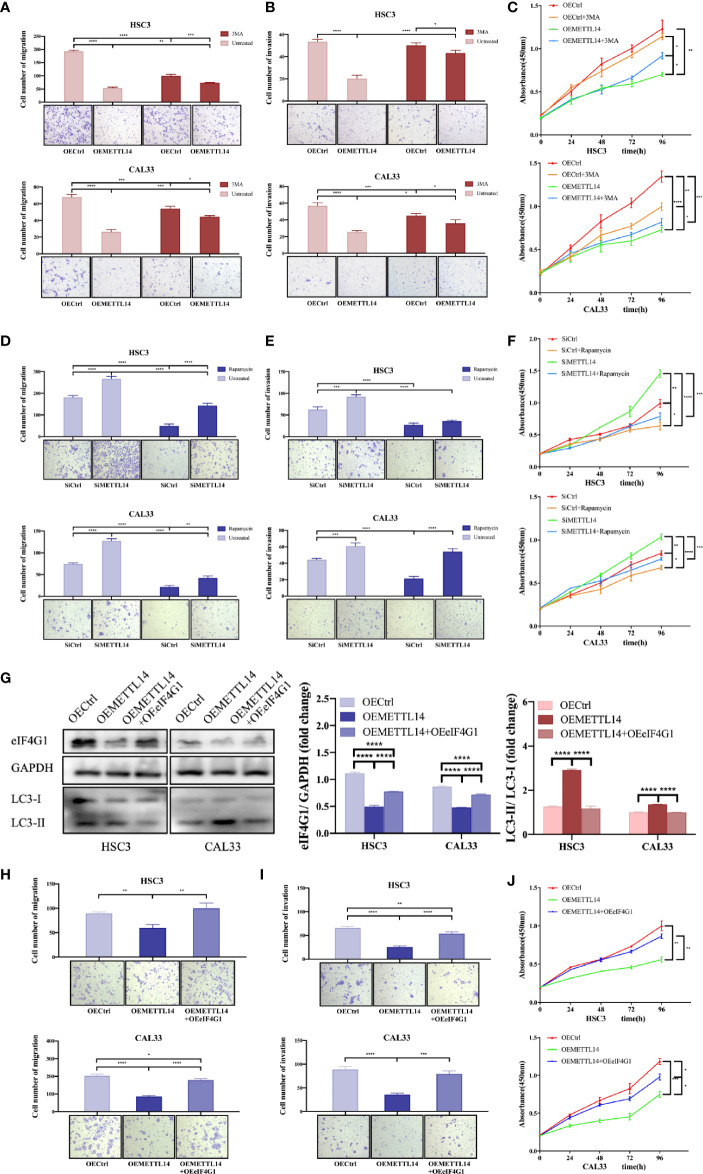
METTL14 regulates autophagy and biological functions of OSCC cells by mediating eIF4G1 expression. Transwell assays were used to detect the migratory and invasive abilities of HSC3 and CAL33 cells (one-way ANOVA). Proliferation of HSC3 and CAL33 cells was detected using CCK8 assays (one-way ANOVA). One group of cells were transfected with OEMETTL14 or OECtrl and cultured with or without 3MA **(A–C)**, while the other group was silenced using siMETTL14 or siCtrl and rescued using rapamycin or not **(D–F)**. **(G)** Western blot was used to compare expression levels of eIF4G1, LC3-I, and LC3-II in HSC3 and CAL33 cells transfected with OECtrl, OEMETTL14, and OEMETTL14+OEeIF4G1. **(H–J)** The migration, invasion, and proliferation of OSCC cells with eIF4G1 overexpression and METTL14 knockdown. (*p* or adjusted-*p* *< 0.05, **< 0.01, ***< 0.001, ****< 0.0001).

In order to study the effect of METTL14 on the biological functions of OSCC cells, we carried out Transwell migration, invasion, CCK-8 proliferation, and rescue experiments. The results showed that METTL14 overexpression inhibited cell migration, invasion, and proliferation, while 3MA partially alleviated the halted biological functions of OSCC by inhibiting autophagy (**Figures 4A**–**C**). METTL14 knockdown and rescue with rapamycin yielded opposing results (**Figures 4D**–**F**). This suggests that the inhibitory effect of METTL14 on OSCC depends on the level of autophagy.

eIF4G1 is a clear negative regulator of autophagy. We performed western blotting to further prove the regulation of METTL14 on eIF4G1 expression at the protein level. The results showed that METTL14 overexpression inhibited eIF4G1 expression and increased autophagy. Overexpressing both METTL14 and eIF4G1 alleviated the increase in autophagy (**Figure 4G**). This suggests that METTL14 promotes autophagy by inhibiting eIF4G1 expression.

Furthermore, we simultaneously overexpressed METTL14 and eIF4G1 to determine whether METTL14 affects the biological function of OSCC cells by mediating the expression of eIF4G1. The results showed that METTL14 overexpression inhibited cell migration, invasion, and proliferation, while eIF4G1 overexpression partially alleviated METTL14-induced inhibition of biological functions in OSCC (**Figures 4H**–**J**). In conclusion, METTL14 promotes autophagy by inhibiting eIF4G1 expression, thereby inhibiting the migration, invasion, and proliferation of OSCC.

### METTL14 Expression *In Vivo*

**Figure 5:**
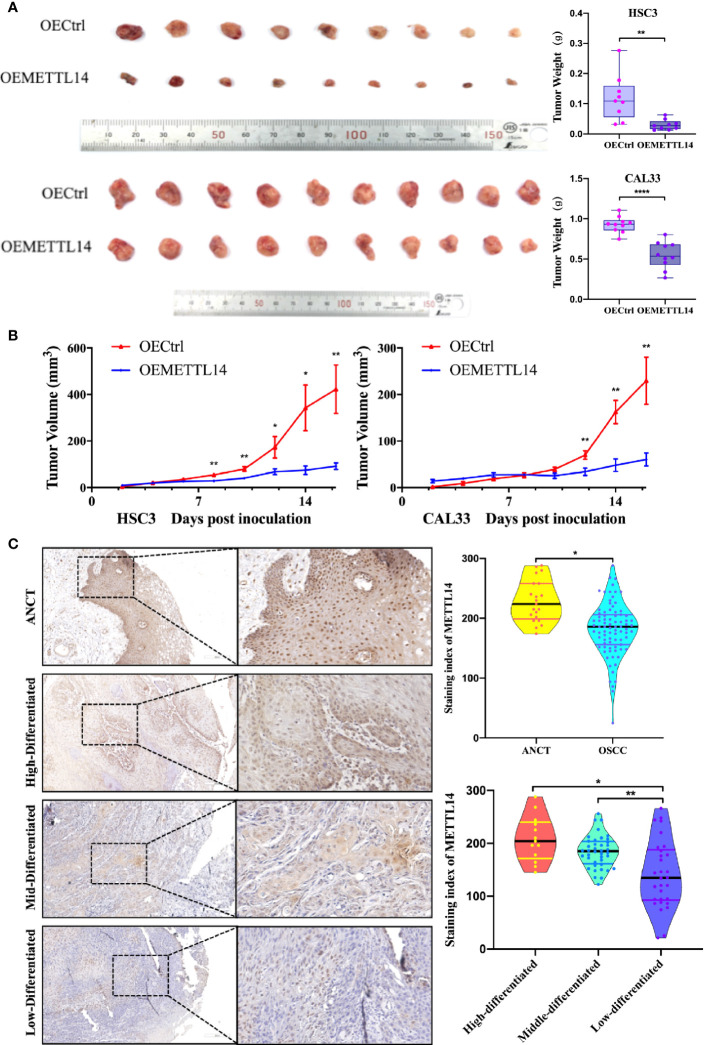
METTL14 expression *in vivo*. **(A)** Growth and weight of tumor xenografts (two weeks) in NOD/SCID mice subcutaneously injected with 5 × 10^7^ HSC3 or CAL33 cells transfected with OECtrl or OEMETTL14. **(B)** Volume of tumor xenografts in NOD/SCID mice calculated every two days [V = (length × width^2^/2)]. **(C)** Representative immunohistochemistry images and correlations of METTL14 in ANCT and OSCC tissues with different pathological differentiations. (*p* or adjusted-*p* *< 0.05, **< 0.01, ***< 0.001, ****< 0.0001).

SPF female NOD/SCID mice (5–6 weeks old) were randomly divided into four groups: HSC3-OECtrl, HSC3-OEMETTL14, CAL33-OECtrl, and CAL33-OEMETTL14 (10 mice in each group). Each mouse was injected with 5 × 10^7^ cells subcutaneously in the inner thigh. Thereafter, water and food intake were normal, and no death occurred. The size of subcutaneous tumours was measured every two days. After two weeks, the mice were sacrificed and the growth curve of the transplanted tumours was created. The results showed that the volume and weight of subcutaneous tumours in the OEMETTL14 group were significantly lower than those in the OECtrl group (**Figure 5A, B**), indicating that METTL14 overexpression can effectively inhibit the growth of OSCC subcutaneous tumours in mice. Immunohistochemistry demonstrated that METTL14 overexpression reduced eIF4G1 abundance (**Supplementary Figure S3**).

To further clarify the expression of METTL14 in tissues and its clinical significance, we evaluated the expression of METTL14 in 82 OSCC tissue samples and 19 ANCT samples. The results showed that the protein expression of METTL14 in OSCC was significantly lower than that in ANCT; the lower the differentiation of tumour cells, the lower the METTL14 expression (**Figure 5C**).

We defined an immunohistochemical score of 150 or more as the high expression group and less than 150 as the low expression group. We further analysed the correlation between METTL14 expression and gender, age, differentiation, T stage, and lymph node metastasis. The results showed that METTL14 expression was negatively correlated with differentiation and T stage (**Table 1**). These results clinically proved that METTL14 expression affected OSCC invasion, metastasis, and prognosis.

**Table 1:** Clinicopathological features and METTL14 expression of OSCC patients.

Clinicopathologicalfeatures	No. of cases	METTL14 expression		*p* value
		High (n)	Low (n)	
**Gender**				
Male	53	17	36	0.1002
Female	29	15	14	
**Age**				
≥55	51	24	27	0.0654
<55	31	8	23	
**Differentiation**				
Well	13	10	3	**0.0032**
Moderate+ poor	69	22	47	
**T stage**				
T_I-II_	72	25	47	**0.0423**
T_III-IV_	10	7	3	
**LN metastasis**				
N^-^	68	27	41	>0.9999
N^+^	14	5	9	

^*^p < 0.05

Bold values indicate statistical differences.

## Discussion

In this study, we used a rapamycin-induced autophagy cell model to carry out *in vitro* experiments. We screened the m^6^A RNA methyltransferase METTL14 as the key enzyme that regulates autophagy levels in OSCC cells. We found that rapamycin regulates the m^6^A modifying enzyme and mediates changes in m^6^A and mRNA expression of related genes. Rapamycin is a new macrolide antibiotic and an anti-cancer drug certified by the FDA (26). Research has shown that rapamycin can prevent or delay the occurrence of tumours, including colon cancer (27), glioblastoma (28), renal cell cancer (29), and ovarian cancer (30). At present, a rapamycin phase III clinical trial has been completed, and its safety and therapeutic effects have been confirmed. A recent clinical study also preliminarily confirmed the efficacy of rapamycin in the treatment of advanced head and neck squamous cell carcinoma (31). The study showed that rapamycin can significantly improve the clinical symptoms of patients within three weeks, and significantly reduce tumour volume. The molecular mechanism through which rapamycin inhibits the growth and proliferation of oral and oropharyngeal squamous cell carcinoma may lie in the inhibition of the PI3K/Akt/mTOR signalling pathway. In this study, we found that rapamycin may inhibit OSCC by regulating the modification of m^6^A to induce autophagy, thereby inhibiting malignant tumour progression. Our results provide a new experimental basis for clinical trials involving rapamycin in the treatment of OSCC and new ideas for targeted autophagy therapy.

Autophagy, a ubiquitous process in eukaryotic cells, not only plays an important role in the renewal, development, and differentiation of normal cell components but also maintains the balance of protein metabolism and DNA stability (32). Autophagy is closely related to the invasive and metastatic mechanisms of tumour cells, such as epithelial mesenchymal transition (EMT) (33), anaerobic glucose metabolism (34), resistance to anoikis (35), and tumour inflammation (36). Studies have confirmed that changes in autophagy impact tumour development (37, 38). Autophagy removes damaged organelles, maintains the stability of intracellular environments, and plays a role in tumour inhibition. More importantly, the loss of autophagy can lead to environmental destabilisation and DNA damage in tumour cells, increase genomic instability under metabolic stress, promote the activation of proto-oncogenes, and increased malignant phenotypes or progression of cancer cells (37, 38). Studies have shown that in many types of tumours, such as breast cancer (39), ovarian cancer (40), and gastric cancer (41), malignant cells usually show lower basal autophagy activity than normal cells. Zhao et al. (42) and other researchers have confirmed that autophagy inhibition can activate the ROS/HO-1 signalling pathway, promote EMT, and enhance the migratory and invasive abilities of ovarian cancer cells. Our previous studies also found that autophagic activity was negatively correlated with the malignant progression of OSCC, while Beclin1, a positive regulator of autophagy, functions to inhibit OSCC (14, 15). In the present study, we found that after autophagy activation, the level of m^6^A and the expression of METTL14 increased, while the expression of FTO decreased. There was no significant change in the expression of METTL3, WTAP, or ALKBH5, suggesting that autophagy may be associated with m^6^A in OSCC. We then studied the effect of METTL14 on the malignant development of OSCC. The results showed that METTL14 inhibits migration, invasion, and proliferation of OSCC by inducing autophagy. These results suggest that METTL14-mediated m^6^A modification and autophagy are closely related to the malignant progression of OSCC.

m^6^A is the most abundant RNA modification form in eukaryotic cells. The methylation of m^6^A mainly occurs in the conservative motif of RRm^6^ACH (G/A/U) [G/A] m^6^AC [U/A/C]) (43), and is enriched in the 3′ UTR region near the termination codon and the long exon (44). The activity of m^6^A is regulated by methyltransferase, demethyltransferase, and m^6^A binding protein, which affect almost all aspects of RNA metabolism, such as RNA splicing, nuclear production, translation, degradation, and interaction between RNA and protein (45, 46). m^6^A methyltransferase is present as a complex, which includes a heterodimer catalytic core formed by METTL3 and METTL14, and regulates the subunit WTAP (47). The demethyltransferase mainly includes FTO and ALKBH5 (48, 49). The m^6^A RNA modification plays a regulatory role in target genes through recognition of the m^6^A binding protein (m^6^A reading protein), while different m^6^A binding proteins may upregulate or downregulate target genes through various mechanisms. The binding proteins of m^6^A mainly include YTH family proteins YTHDF1/2/3, YTHDC1/2, and IGF2BP1/2/3.

Studies have shown that m^6^A affects the malignant progression of tumours by regulating the proliferation, differentiation, and apoptosis of tumour cells. Downregulation of METTL3 or METTL14 can promote proliferation and self-renewal of glioblastoma stem cells by reducing the m^6^A level on *ADAM19* mRNA, which enhances its stability and finally promotes the occurrence of glioblastoma (17). METTL14 can promote the maturation of miR-126, a tumour suppressor, by promoting the combination of pri-miR126 and DGCR8 modified with m^6^A. The lack of METTL14 in hepatocellular carcinoma can inhibit the production of miR126 and induce metastasis (18). Furthermore, METTL14 is highly expressed in acute myelocytic leukaemia (AML) and can promote the occurrence of AML by inhibiting the degradation of *MYB* and *MYC* mRNA, promoting its translation and maintaining the self-renewal of leukaemia stem cells (19). Meanwhile, FTO expression in AML patients is increased. FTO inhibited the expression of *ASB2* and *RARA* mRNA by downregulating m^6^A modification of target genes, inhibited the induction and differentiation of trans-retinoic acid on AML cells, and promoted AML development (20). On the other hand, R-2-hydroxyglutaric acid (R-2HG) can stabilize the expression of *MYC*/*CEBPA* mRNA by inhibiting FTO demethylation and blocking mRNA degradation mediated by the m^6^A binding protein YTHDF2, thus inhibiting the proliferation of AML cells (50). The above studies indicated that m^6^A modification can regulate the malignant progression of tumours by affecting mRNA metabolism, but there are great differences in the regulation of various tissues and cells, which may be related to different m^6^A binding proteins regulating different target genes.

m^6^A modification mainly regulates gene expression, which is mediated by RNA binding proteins (16). There are many m^6^A binding proteins, which are widely targeted. For example, the HNRNP family can regulate the variable splicing of RNA (51). IGF2BP1/2/3 are involved in the regulation of RNA translation (51) and YTHDC1 is involved in nucleic acid transport (52), while HuR can enhance RNA stability (53). YTHDF2 and YTHDC2 can promote RNA degradation (24, 25). YTHDF2, located in the cytoplasm, can bind to the m^6^A modification site by direct recognition, and then activate the downstream regulatory pathway through interaction with other proteins (25). A study showed that YTHDF2 can promote the degradation of *PD1* mRNA in melanoma (54), and *LHPP* and *NKX3-1* in prostate cancer (55). Our study shows that YTHDF2 can promote the degradation of *eIF4G1* mRNA, but also functions through other mechanisms. A study reported that under heat stress, YTHDF2 can enter the nucleus, inhibit FTO demethylation of the 5′ UTR region of mRNA, then activate the non-5′ cap dependent translation initiation mechanism, and thus promote the translation of mRNA (24). Therefore, different binding proteins may have different functions, and the same binding protein may also have different functions in different intracellular environments. In addition, the complexity of m^6^A modification in the regulation of mRNA is also reflected in its target site. Several studies suggest that m^6^A is mainly enriched in the 3′ UTR region and near the stop codon of polyadenylated mRNA (56). However, a previous study found that the m^6^A site that mediates *snail* mRNA translation in cancer cells is located in the CDS region rather than the 3′ UTR region (57). Our sequencing and dual luciferase reporter gene experiments also reached similar results. The m^6^A site of YTHDF2 binding to *eIF4G1* mRNA was located in the CDS region. Therefore, the binding sites differ amongst different genes and binding proteins.

**Figure 6:**
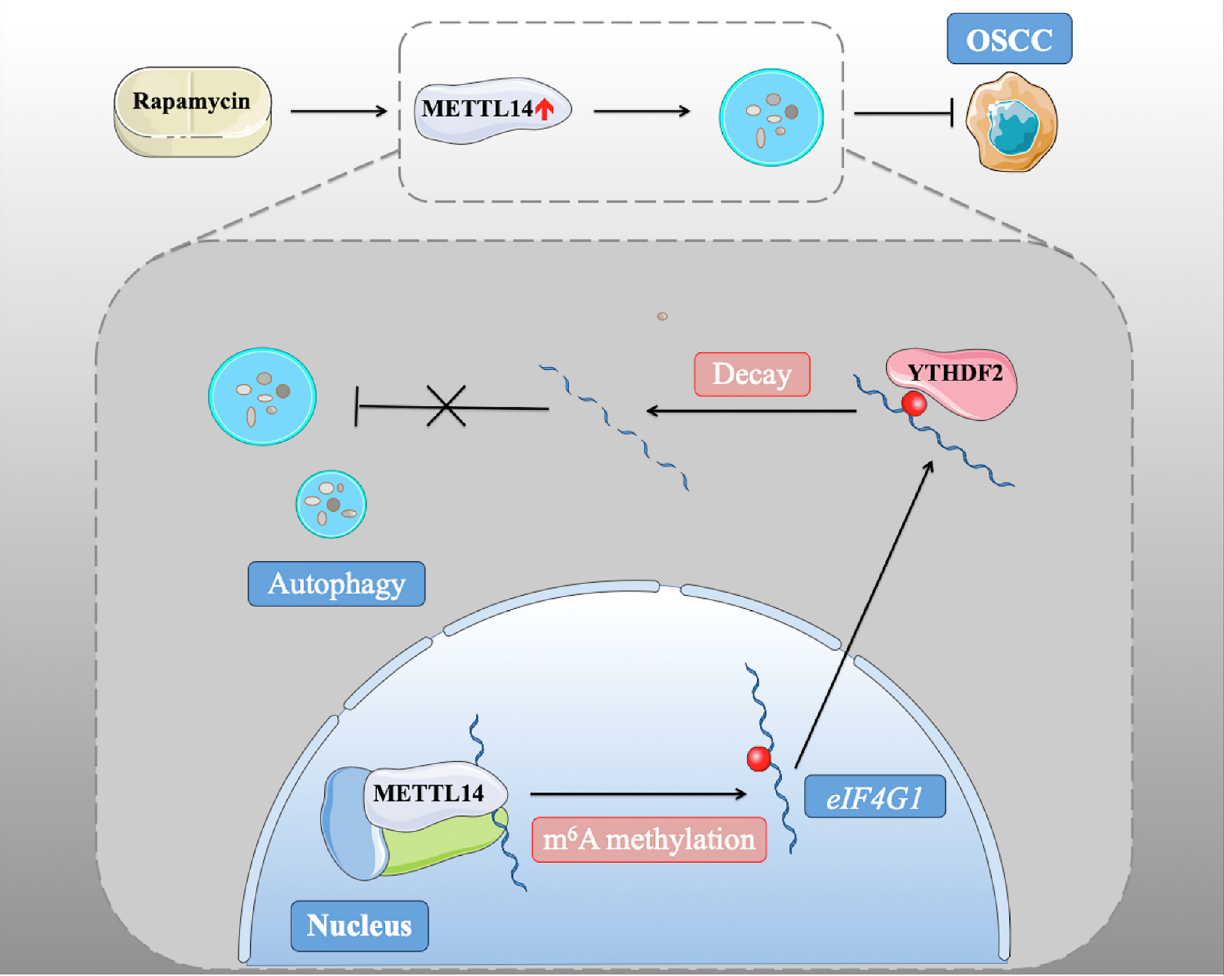
METTL14 down-regulated eIF4G1 mRNA stability in a YTHDF2-mediated m^6^A manner.

In conclusion, we found that METTL14 plays a conservative and important role in promoting autophagy and inhibiting tumorigenesis through its methylation function (**Figure 6**). These results were confirmed in xenograft nude mice and human patient samples, emphasizing the interaction between m^6^A methylation and autophagy in oncogenesis and the malignant characteristics of OSCC. Our study identified the mechanism by which rapamycin affects autophagy *via* regulating METTL14, which provides a new idea for a potential targeted therapy for OSCC.

## Data Availability Statement

The data presented in the study are deposited in the GEO repository, accession number GSE186581.

## Ethics Statement

The studies involving human participants were reviewed and approved by the ethics committee of the Hospital of Stomatology, Sun Yat-Sen University. The patients/participants provided their written informed consent to participate in this study. The animal study was reviewed and approved by Institutional Animal Care and Use Committee of Sun Yat-sen University, Guangzhou, China.

## Author Contributions

FW and YuZ conceived and designed the experiments. FW, YuZ, HC, WW, YL, and YaZ performed the experiments. FW, YuZ, HC, HL collected and analyzed the data. FW wrote the original manuscript. CW and JH reviewed and edited the manuscript. All authors contributed to the article and approved the submitted version.

## Funding

This work was supported by the National Natural Science Foundation of China (Grant Nos. 81874128 and 82072994) and fundamental research program funding of Ninth People's Hospital affiliated to Shanghai Jiao Tong university School of Medicine (Grant No. JYZZ158).

## Conflict of Interest

The authors declare that the research was conducted in the absence of any commercial or financial relationships that could be construed as a potential conflict of interest.

## Publisher’s Note

All claims expressed in this article are solely those of the authors and do not necessarily represent those of their affiliated organizations, or those of the publisher, the editors and the reviewers. Any product that may be evaluated in this article, or claim that may be made by its manufacturer, is not guaranteed or endorsed by the publisher.
