# Supplementary figures and images for: N6-Methyladenosine Methyltransferase METTL14-Mediated Autophagy in Malignant Development of Oral Squamous Cell Carcinoma
Source: Front Oncol. 2021 Nov 24;11:738406. doi: 10.3389/fonc.2021.738406 (PMC8652297; doi:10.3389/fonc.2021.738406)

Fig. S1

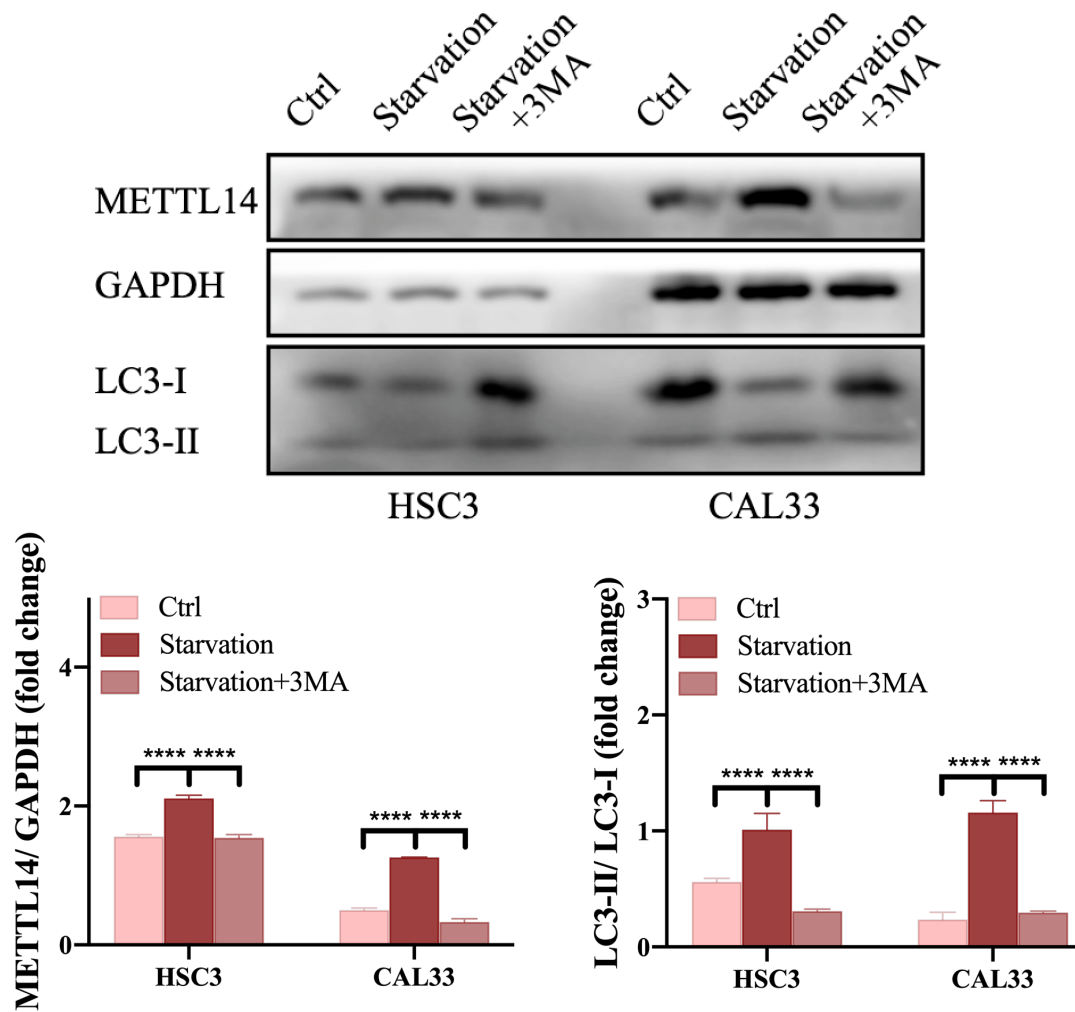

Fig. S2

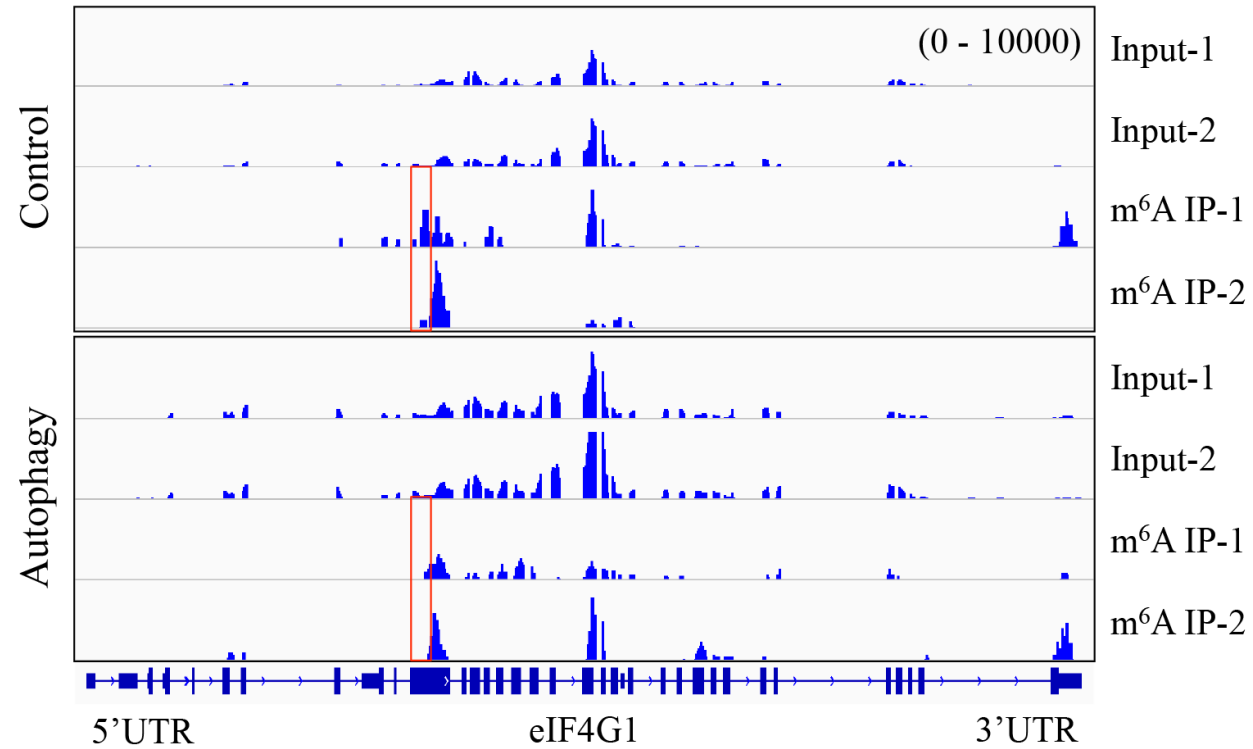

Fig. S3

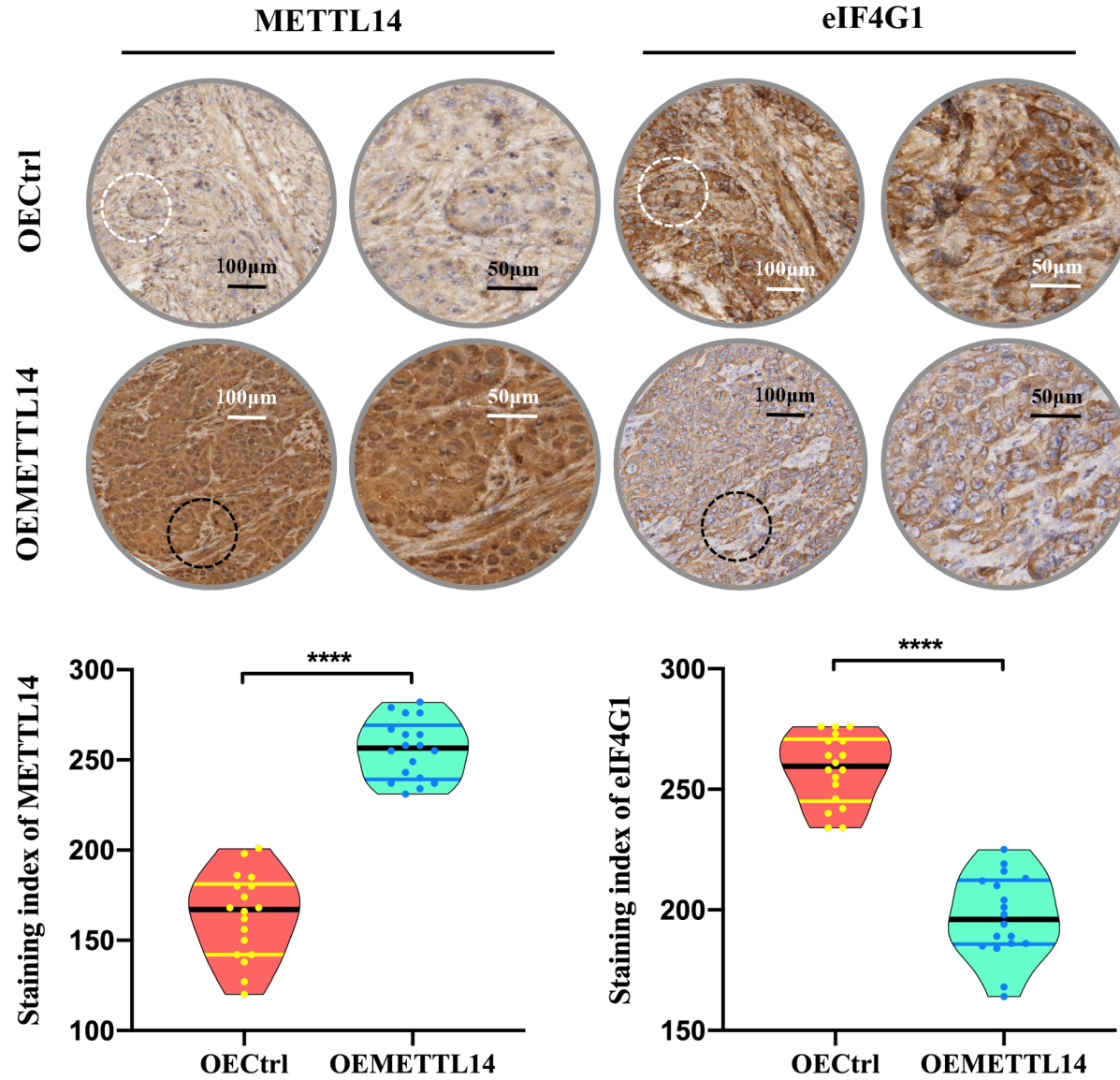

Supplement: Supplementary file 1 [file DataSheet_1.pdf]
